# Electrochemically Synthesized Silver Nanoparticles Are Active Against Planktonic and Biofilm Cells of *Pseudomonas aeruginosa* and Other Cystic Fibrosis-Associated Bacterial Pathogens

**DOI:** 10.3389/fmicb.2018.01349

**Published:** 2018-07-05

**Authors:** Arianna Pompilio, Cristina Geminiani, Domenico Bosco, Rosalba Rana, Antonio Aceto, Tonino Bucciarelli, Luca Scotti, Giovanni Di Bonaventura

**Affiliations:** ^1^Department of Medical, Oral and Biotechnological Sciences, “G. d’Annunzio" University of Chieti-Pescara, Chieti, Italy; ^2^Center of Excellence on Aging and Translational Medicine (CeSI-MeT), Chieti, Italy; ^3^Department of Biomorphological Science, Molecular Genetic Institute, Italian National Research Council, Chieti, Italy; ^4^Department of Medicine and Science of Aging, “G. d’Annunzio” University of Chieti-Pescara, Chieti, Italy

**Keywords:** silver nanoparticles, cystic fibrosis, *Pseudomonas aeruginosa*, biofilm, antibacterial activity

## Abstract

A novel, electrochemically synthesized, silver nanoparticles (AgNPs) formulation was evaluated *in vitro* against *Pseudomonas aeruginosa*, *Burkholderia cepacia*, *Stenotrophomonas maltophilia*, and *Staphylococcus aureus* strains from cystic fibrosis (CF) patients. AgNPs were particularly active against *P. aeruginosa* and *B. cepacia* planktonic cells (median MIC: 1.06 and 2.12 μg/ml, respectively) by a rapid, bactericidal and concentration-dependent effect. AgNPs showed to be particularly effective against *P. aeruginosa* and *S. aureus* biofilm causing a viability reduction ranging from 50% (1×MIC) to >99.9% (4×MIC). Electron microscopy showed that AgNPs deconstruct extracellular matrix of *P. aeruginosa* biofilm, and accumulate at the cell surface causing cell death secondary to membrane damage. Compared to Tobramycin, AgNPs showed comparable, or even better, activity against planktonic and biofilm *P. aeruginosa* cells. AgNPs at concentrations effective against *B. cepacia* and *P*. *aeruginosa* were not toxic to *G. mellonella* larvae. Our silver-based formulation might be an alternative to antibiotics in CF patients. Further *in vitro* and *in vivo* studies are warranted to confirm this therapeutic potential.

## Introduction

Cystic fibrosis patients are prone to chronic infection of the respiratory tract, which ultimately leads to pulmonary failure, the primary cause of death in this patient population (Ciofu et al., 2013). CF patients have a peculiar set of bacterial pathogens that are frequently acquired in an age-dependent sequence (Cystic Fibrosis Foundation, 2016). *Staphylococcus aureus* and *Pseudomonas aeruginosa* are the most prevalent respiratory pathogens, respectively, in younger and adult CF patients (Cox et al., 2010). Other pathogens, such as *Burkholderia cepacia* complex and *Stenotrophomonas maltophilia*, are less frequently recovered but particularly troublesome in these patients due to their multidrug-resistant phenotypes and can cause a severe decline in lung function (Speert et al., 2002; Berdah et al., 2018). The frequency of respiratory tract infection by these species increases with patient age, posing a significant health risk to CF patients surviving to adulthood. The antibiotic therapy is, in fact, hardly affected by broad-spectrum resistance, both constitutive and inducible, exhibited by most strains (Kerem, 2017).

Most of CF pathogens also are highly adapted to the CF pulmonary environment, and one of the key strategic adaptation mechanisms includes the formation of biofilms, cellular aggregations embedded in EPS to protect bacteria from the antibiotic therapy and host immunity (de la Fuente-Núñez et al., 2013; Olsen, 2015). This scenario is further complicated by the evidence that at the site of infection pathogens grow in highly viscous sputum whose composition (extracellular-DNA, lipids, proteins, etc.) affects both delivery and functionality of antibiotics (Hunt et al., 1995).

The rise of multidrug-resistant bacteria and the difficulty of treating chronic biofilm-mediated infections have prompted a renewed need for novel antimicrobial agents (de la Fuente-Núñez et al., 2013; Smith et al., 2017). In recent years, several studies have reported metallic nanomaterials as a promising alternative to antibiotics because of their relevant bactericidal effects, thus suggesting a high potential in medical devices, burn dressings, water treatment and food preservation (Vimbela et al., 2017). In particular, now as antibiotic-resistant bacterial strains continue to emerge and rise in number, silver has been introduced as a promising material in the development of new bactericides (Blaskovich et al., 2017; Johnson et al., 2017). Silver nanoparticles (AgNPs) have shown novel antimicrobial activity to a wide range of microorganisms due to their high surface area to volume ratio and their unique chemical and physical properties when compared to the properties of their bulk form.

The antibacterial potential of AgNPs has been reported against both Gram-negative and Gram-positive bacteria (Sondi and Salopek-Sondi, 2004; Jain et al., 2009), and six clinical trials are actually ongoing, two of which are focused on anti-biofilm activity^1^. Nevertheless, their activity against CF pathogens has not yet received considerable attention (Habash et al., 2014; Zhang et al., 2015).

Therefore, in the present study for the first time we evaluated the activity of a novel AgNPs formulation against multidrug-resistant *P. aeruginosa*, *B. cepacia*, *S. maltophilia*, and *S. aureus* strains recovered from patients with CF. Particularly, AgNPs were assayed for *in vitro* activity against both planktonic cells and mature biofilm, and for cytotoxic potential in the invertebrate wax moth larva model. Finally, morphological changes induced in *P. aeruginosa* biofilm were monitored by transmission electron microscopy (TEM). In the case of *P. aeruginosa*, AgNPs were evaluated comparatively to Tobramycin.

## Materials and Methods

### Nanoparticles Generation and Chemicals

AgNPs were synthesized by a new electronic device using an electrochemical method we recently described (Scotti et al., 2017). Briefly, an electrolytic cell containing silver plate electrodes (75 × 28 × 0.5 mm) worked in a sacrificial mode (electrodes interdistance 10 mm). A power supply and a home-made electronic board were employed to generate 200 V electrical current and 0.5 Hz square wave frequency. AgNPs were produced by red-ox reactions occurring during the electrochemical process. The following parameters were monitored and automatized: constant voltage (V), current (A) and total electrical power (W); temperature; time reaction (10 min).

UV–vis spectroscopy (Jasco 7800; Jasco, Easton, MD, United States), TEM, FE-SEM and dynamic laser light scattering (DLS) were performed to characterize AgNPs for size, shape, and morphology. TEM images were taken, at 75 kV, using ZEISS 109 equipped with Gatan-Orius SC200W-Model 830.10W TEM CCD Camera, whereas FE-SEM analysis was performed by Sigma 300 Zeiss equipped with elemental microanalysis apparatus (Quantax-200 Bruker). DLS was performed by 90Plus/BI-MAS equipped with ZetaPALS Particle Sizing Software ver 3.86 (Brookhaven Instruments Corp., Holtsville, NY, United States).

Tobramycin powder was purchased from Sigma-Aldrich (Milan, Italy), prepared as a stock solution in distilled water at 5 mg/ml, 0.2 μm-filtered, and then stored at −80°C until use.

Cation-adjusted Mueller-Hinton broth (CAMHB), Tryptic Soy broth (TSB), Tryptic Soy agar (TSA), and Mueller-Hinton agar (MHA) were purchased from Oxoid (Garbagnate M.se, Milan, Italy) and prepared according to the manufacturer’s indications.

### Bacterial Strains

Three strains each of *Pseudomonas aeruginosa* (Pa14, AC12A, and DIN1), *Stenotrophomonas maltophilia* (SanG2010, DAT7, and AC8), *Burkholderia cepacia* (Bc6, Bc11, and Bc23), and *Staphylococcus aureus* (Sa1, Sa2, and Sa3) were tested. Kirby–Bauer disk-diffusion test showed that all strains are multidrug-resistant, according to the definition proposed by Magiorakos (Magiorakos et al., 2012) (Supplementary Table S1). Each strain was isolated from respiratory specimens collected from a single patient at the CF Microbiology Laboratory, “Bambino Gesù” Children Hospital, Rome. Strains were stored at −80°C in a Microbank system (Biolife Italiana S.r.l., Milan, Italy) and subcultured in TSB, then twice on MHA prior to the use in this study.

### Standardization of the Bacterial Inoculum

Some colonies from an overnight 37°C growth onto MHA (*P. aeruginosa, S. maltophilia*, and *S. aureus*) or TSA (*B. cepacia*) were resuspended in CAMHB to an OD measured at 550 nm (OD_550_) of 1.0 (corresponding to 1−5×10^9^ CFU/ml). This standardized bacterial suspension was then diluted (vol/vol) accordingly to use.

### AgNPs and Tobramycin Activity Against Planktonic Bacteria

MICs and MBCs of both AgNPs and Tobramycin were determined by microdilution technique, in accordance with CLSI guidelines (Clinical Laboratory Standards Institute [CLSI], 2016), with some modifications. Briefly, serial two-fold dilutions of AgNPs (ranging from 17 to 0.033 μg/ml, corresponding to 0.7 × 10^9^ to 4.5 × 10^7^ nanoparticles/ml) and Tobramycin (256–0.5 μg/ml) were prepared in CAMHB at a volume of 100 μl/well in 96-well microtiter plates (Kartell LabWare; Noviglio, Italy). Each well was then inoculated with 5 μl of the standardized inoculum, corresponding to a final test concentration of about 0.5−1 × 10^5^ CFU/well. After incubation at 37°C for 20 h, the MIC was read as the lowest concentration of the test agent that completely inhibited visible growth. To measure the MBC, 100 μl of broth from clear wells were plated on TSA plates and incubated at 37°C for 24 h. MBC was defined as the lowest concentration of the test agent killing of at least 99.99% of the original inoculum.

### Time-Killing Assay

Kinetics of AgNPs and Tobramycin activity was evaluated by using the broth macrodilution method. Briefly, the standardized inoculum (1 × 10^5^ CFU/mL) was exposed to the test agent at 1×, 2×, and 4×MIC in 10 ml CAMHB, and incubated at 37°C. After 1, 2, 3, 4, 5, 6, and 24 h of incubation, aliquots of each sample underwent viable cell counts onto TSA. Killing curves were obtained by plotting the log CFU/mL versus time.

### Screening for Biofilm Formation

Bacteria were grown overnight in TSB, adjusted with fresh TSB to an OD_550_ of 1.00 (corresponding to about 1 × 10^9^ CFU/ml), and 200 μl of 1:100 diluted inoculum were dispensed to each well of a sterile flat-bottom polystyrene tissue culture 96-wells microtiter (Corning; Turin, Italy). Control wells contained medium alone. After 24-h incubation at 37°C, non-adherent bacteria were removed by washing twice with 200 μl sterile phosphate-buffered saline pH 7.3 (PBS) (Sigma-Aldrich), and biofilm biomass was then evaluated by crystal violet assay and measured as the OD at 492 nm (OD_492_) (Pompilio et al., 2015). Considering a low cut-off (OD_c_) represented by 3 × SD above the mean OD of control wells, strains were classified as (Stepanović et al., 2007): no biofilm producer (OD ≤ OD_c_), weak biofilm producer (OD_c_ < OD ≤ 2 × OD_c_), moderate biofilm producer (2 × OD_c_ < OD ≤ 4 × OD_c_), or strong biofilm producer (4 × OD_c_ < OD).

### AgNPs and Tobramycin Activity Against Preformed Biofilms

The antibiofilm activity of AgNPs and Tobramycin was evaluated against *P. aeruginosa* DIN1, *S. maltophilia* Sang2010, *B. cepacia* Bc23 and *S. aureus* Sa2 strains selected because of strong biofilm producers. In the case of *P. aeruginosa* DIN1, AgNPs antibiofilm activity was assessed comparatively to Tobramycin. Biofilms were grown for 24 h at 37°C in each well of a 96-well flat-bottom polystyrene tissue-treated microtiter plate, then exposed to 200 μl of test agent-containing CAMHB (each prepared at concentrations equal to 1×, 2×, and 4×MIC). After incubation at 37°C for 24 h, non-adherent bacteria were removed by washing once with 200 μl sterile PBS (pH 7.3), and biofilm samples were scraped with a pipette tip following 5-min exposure to 100 μl trypsin-ethylenediaminetetraacetic acid 0.25% (Sigma-Aldrich). The cell suspension was vortexed at maximum speed for 1 min to break up bacterial clusters and then underwent to viable cell counts on MHA (*P. aeruginosa*, *S. maltophilia*, and *S. aureus*) or TSA (*B. cepacia*) plates.

### Electron Microscopy

The effects caused by AgNPs on the ultrastructure of an established biofilm were assessed by TEM choosing *P. aeruginosa* DIN1 strain because the highest biofilm producer among strains considered. One-day biofilm was grown in CAMHB as previously described, and then samples were exposed for further 24 h to AgNPs or Tobramycin at 4×MIC in CAMHB, or to CAMHB only (controls). At the end of exposure samples were washed twice with PBS, scraped, centrifuged (16400 rpm, 10 min, 4°C), then the pellet was fixed in 2.5% glutaraldehyde (Sigma-Aldrich) (vol/vol) in 0.1 M sodium cacodylate buffer (pH 7.4) for 2 h, and post-fixed in tetroxide osmium. After being washed, the samples were dehydrated in a series of aqueous ethanol solutions (30–100%) and embedded in Spurr resin. Ultrathin sections (60–80 nm) were mounted on 200-mesh nickel grids, stained with or without uranyl acetate and lead citrate, and observed with a TEM (ZEISS 109 equipped with Gatan-Orius SC200W-Model 830.10W TEM CCD Camera) microscope.

### *In Vivo* Toxicity Assay

Toxicity of AgNPs and Tobramycin was comparatively assessed in the wax moth larva *Galleria mellonella* (Desbois and Coote, 2012). No ethical approval was required for the study because there was no use of a mammalian model of infection and animal house. For each group, 20 larvae weighing 250–350 mg were injected using Hamilton syringe, directly into the hemocoel via the right proleg, with 10 μl containing test agent at desired concentration (AgNPs: 6.8 and 3.4 μg/ml; Tobramycin: 4 μg/ml). Two control groups of larvae were considered: (i) inoculated with distilled water only, to simulate trauma associated with nanoparticles administration; (ii) not inoculated. Larvae were incubated in the dark at 37°C in Petri dishes, and the number of dead caterpillars was scored every 24 h until 96 h, considering as dead those non-responsive to touch.

### Statistical Analysis

All experiments were performed at least in triplicate and repeated on two different occasions. Statistical analysis was performed using Prism 6 (version 6.01; GraphPad Software Inc., La Jolla, United States), considering *p*-values lower than 0.05 as significant. Differences were measured by chi-square test (frequencies), ANOVA followed by Tukey’s multiple comparison *post hoc* test (viable cell count), or Log-rank (Mantel-Cox) test (survival curve). Differences between MIC values were considered as significant for discrepancies ≥2 log_2_ concentration steps.

## Results

### AgNPs Characterization

**FIGURE 1:**
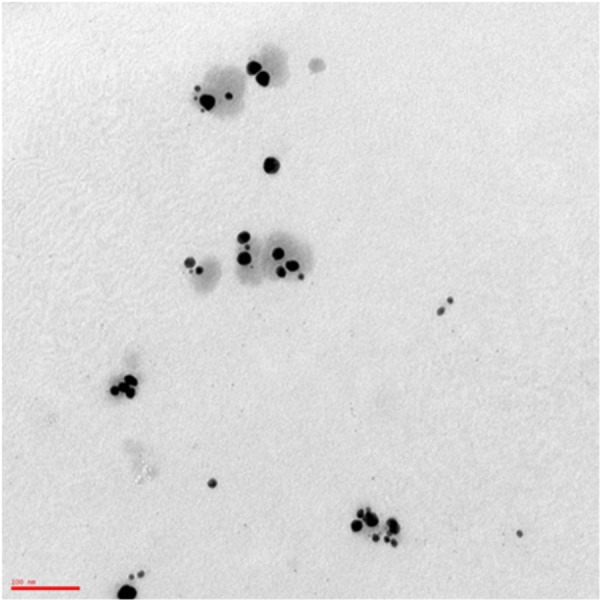
Transmission electron micrograph of AgNPs. A drop of 1:50 diluted stock solution of AgNPs was allowed to evaporate onto 300 mesh formvar coated nickel grids, then TEM image was taken at 75 kV by ZEISS 109 microscope. The image shows quasi-spherical AgNPs with a mean geometric diameter of 43.9 nm. Bar: 100 nm. Magnification: 85.000×.

The yield of AgNPs obtained through electrochemical synthesis was 8.53 mg (Absorbance at 406 nm was 1.79). The product was a yellow solution, odorless with pH of 7–8, and characterized by good stability (6 months) according to the zeta-potential ζ value Z-potential value (−51.5 ± 2.5 mV) and DLS parameters (tracking size, polydispersity index, viscosity, correlation function) (Supplementary Data Sheets 1–4). TEM analysis revealed AgNPs population mostly consists of quasi-spherical uncoated and not-aggregated particles (**Figure 1**) with an average diameter of 55.6 ± 2.9 nm (D10: 26.3 ± 3.7 nm; D50: 30.0 ± 0.0 nm; D90: 81.7 ± 10.3 nm) as assessed by DLS. FE-SEM microanalysis indicated the presence of silver, thus confirming the purity of the synthesized product. A stock solution of AgNPs was prepared in distilled sterile water at a concentration of 34.1 μg/ml, corresponding to 1.47 × 10^9^ NPs/ml.

### AgNPs Show a Rapid Bactericidal Effect Against Gram-Negative Planktonic Cells

To evaluate the antibacterial susceptibility of CF pathogens toward AgNPs, MIC, and MBC were determined by broth microdilution method, and results are shown in **Table 1**.

**Table 1:** *In vitro* activities of AgNPs and Tobramycin against planktonic cells of *P. aeruginosa, B. cepacia, S. maltophilia*, and *S. aureus* strains from CF patients.

	AgNPs		Tobramycin	
Bacterial strains	MIC	MBC	MIC	MBC
***P. aeruginosa***				
Pa14	1.06	2.125	1	1
AC12A	1.06	2.125	4	4
DIN1	4.25	4.25	2	2
***B. cepacia***				
Bc6	2.125	4.25	>256	NT^∗^
Bc11	2.125	4.25	>256	NT
Bc23	1.06	2.125	>256	NT
***S. maltophilia***				
DAT7	4.25	4.25	>256	NT
SanG2010	4.25	4.25	>256	NT
AC8	4.25	4.25	>256	NT
***S. aureus***				
Sa1	8.5	17.0	NT	NT
Sa2	8.5	8.5	NT	NT
Sa3	8.5	8.5	NT	NT
**Total (*n* = 12)**				
MIC_50_^∗^	4.25			
MIC_90_^∗^	8.5			
MBC_50_^†^		4.25		
MBC_90_^†^		8.5		

*MIC and MBC values were obtained by broth microdilution technique, in accordance with CLSI guidelines (2016), and expressed as μg/ml.*^∗^*MIC_50_ and MIC_90_ values are the lowest concentration of the test agent at which 50 and 90% of the isolates were inhibited, respectively. aaaMBC_50_ and MBC_90_ are the minimum concentration killing 50 and 90% of tested strains, respectively. NT, not tested.*

All strains were inhibited by the concentrations of AgNPs tested. The MIC_50_ and MIC_90_ values for the entire panel of 12 strains were 4.25 and 8.5 μg/ml, respectively. Three strains (25%) were inhibited by the lowest concentration of AgNPs tested (1.06 μg/ml), and 2 strains required a concentration of 2.125 μg/ml for inhibition.

Activities were not comparable across the species tested. The activity exhibited by AgNPs against *P. aeruginosa* was comparable to that observed against *B. cepacia* strains (median MIC: 1.06 vs. 2.125 μg/ml, respectively), but significantly higher with respect to *S. maltophilia* and *S. aureus* strains (MIC: 4.25 vs. 8.5 μg/ml, respectively). Compared to Tobramycin, AgNPs showed comparable (against Pa14 and DIN1 strains) or even better (against AC12A strain) activity against *P. aeruginosa* strains.

All MBCs were within 1 log_2_-dilution of the respective MICs. Different susceptibility patterns were observed between AgNPs and Tobramycin.

**FIGURE 2:**
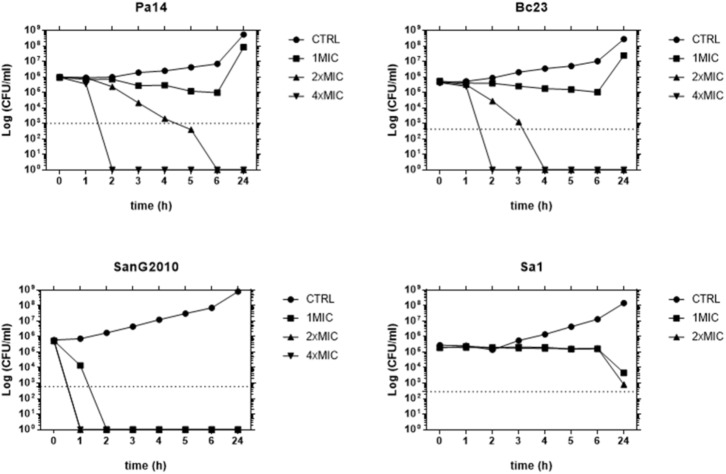
Time-kill study of AgNPs activity. Each strain was exposed to concentrations of AgNPs equal to 1×, 2×, and 4×MIC. After 1, 2, 3, 4, 5, 6, and 24-h of incubation at 37°C, population size was measured by viable bacterial counts. The dotted line indicates a 99.9% killing (10^3^-fold reduction of viability).

A time-kill study performed against strains representative for each species tested (Pa14, Sa1, Bc23, and SanG2010) showed that AgNPs caused a time- and concentration-dependent killing against all species, although significant differences were observed with regard to both the extent and the rapidity of the antibacterial effect (**Figure 2**). AgNPs showed the highest activity against *S. maltophilia* causing a 99.9% decrease in bacterial viability within 90 min at a concentration equals to MIC and complete killing within 1 h at concentrations two and four times greater than the MIC. A bactericidal effect was observed also against both *P. aeruginosa* and *B. cepacia*, although at higher concentrations. Contrarily, only a bacteriostatic effect was observed against *S. aureus*, regardless of concentration tested.

### AgNPs Reduce Viability of Mature *P. aeruginosa* Biofilms in a Dose-Dependent Manner

**FIGURE 3:**
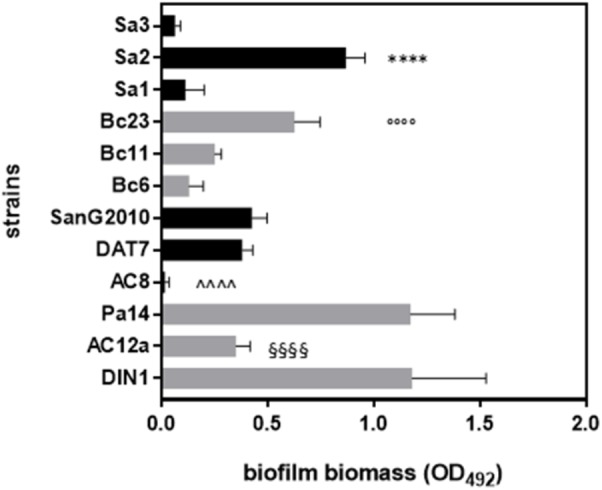
Biofilm formation by 12 bacterial strains from CF patients. Biofilm was allowed to form, after 24 h incubation at 37°C, in polystyrene 96-well microtiter, then biofilm biomass was measured (OD_492_) following crystal violet stain assay. Results are shown as mean values + SDs (*n* = 8). ANOVA + Tukey’s multiple comparison post-test: ^∗∗∗∗^*p* < 0.0001 vs Sa1 and Sa3; ^∘∘∘∘^*p* < 0.0001 vs. Bc6 and Bc11; ^∧∧∧∧^*p* < 0.0001 vs. SanG2010 and DAT7; ^§§§§^*p* < 0.0001 vs. Pa14 and DIN1.

All strains were first screened for biofilm forming ability onto polystyrene by crystal violet assay, and results are shown in **Figure 3**. All strains tested were biofilm-producers, although with striking differences in biofilm formation efficiency. The AgNPs activity against preformed biofilm was assessed toward the best biofilm-producer strain of each species: *P. aeruginosa* DIN1, *S. maltophilia* SanG2010, *B. cepacia* Bc23, and *S. aureus* Sa2. All these strains could be classified as “strong” biofilm producers, according to Stepanović et al. (2007).

**FIGURE 4:**
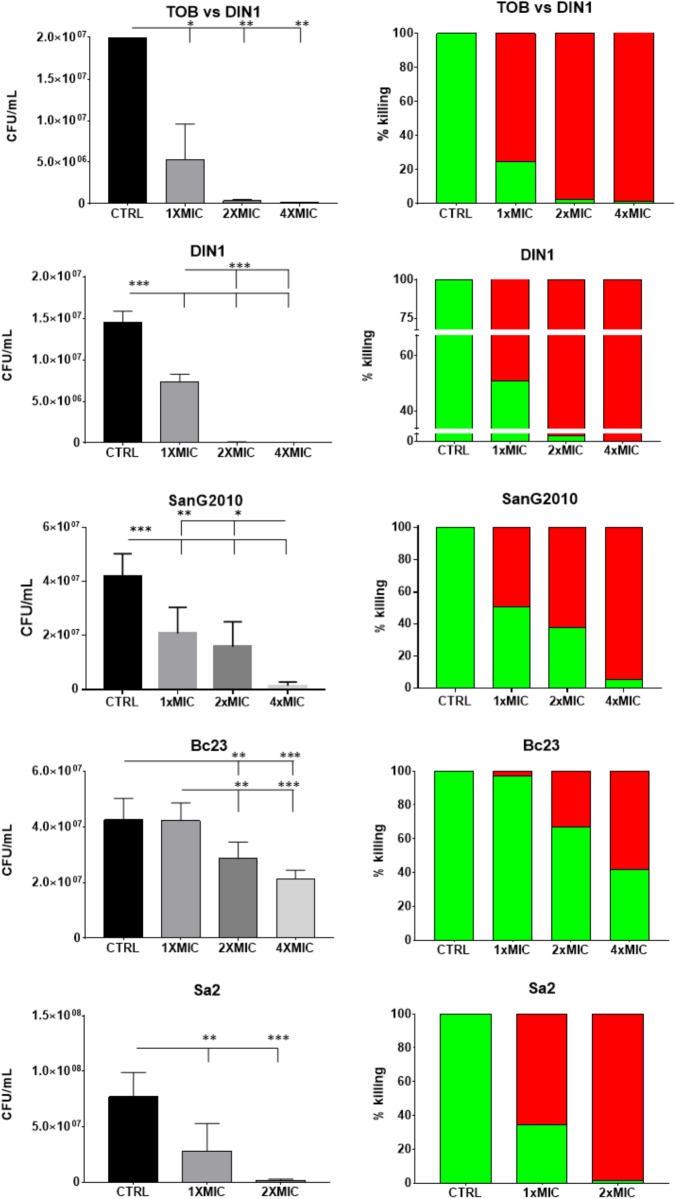
*In vitro* activity of AgNPs against preformed biofilm: viable cell count. Biofilm samples by *P. aeruginosa* DIN1, *S. maltophilia* SanG2010, *B. cepacia* Bc23 and *S. aureus* Sa2 were allowed to form for 24 h in 96-well microtiter plate, then exposed for further 24 h to AgNPs, and also to Tobramycin in the case of *P. aeruginosa* DIN1 strain (TOB vs. DIN1), at concentrations equal to 1×, 2×, and 4×MIC. Biofilm formed by *S. aureus* Sa2 strain was exposed to 1× and 2×MIC since MIC value was 1:2. Control biofilm was not exposed to test agent (CTRL). Results are expressed as CFU/ml (mean + SD; *n* = 6) (left column), and as percentage of killing (right column; the proportion of dead and live cells after exposure was indicated by red and green bars, respectively). ANOVA + Tukey’s multiple comparison post-test: ^∗^*p* < 0.05, ^∗∗^*p* < 0.01, ^∗∗∗^*p* < 0.001.

The effects of AgNPs on already established and mature biofilms are summarized in **Figure 4** and evaluated comparatively to Tobramycin only in the case of *P. aeruginosa*. Exposure of biofilm formed by *P. aeruginosa* DIN1 strain to AgNPs or Tobramycin significantly reduced biofilm viability compared to unexposed controls regardless of concentration tested. However, only AgNPs showed a dose-dependent effect and caused biofilm eradication at concentration equals to 4×MIC, whereas Tobramycin showed a concentration-independent effect causing a maximum reduction of 98.6 ± 1.4% of biofilm viability at 4×MIC. No statistically significant differences in killing activity were found between AgNPs and Tobramycin tested at 1× and 2×MIC.

With regard to the species tested, AgNPs showed to be more active against preformed biofilm by *P. aeruginosa* DIN1 and *S. aureus* Sa2, followed by *S. maltophilia* SanG2010, and then the less susceptible *B. cepacia* Bc23. A comparable maximum killing rate was in fact achieved already at 2×MIC for *P. aeruginosa* DIN1 and *S. aureus* Sa2 (99.5 ± 0.5% and 98.2 ± 0.5%), but it required 4×MIC for *S. maltophilia* SanG2010 (94.7 ± 0.5%). AgNPs were significantly less active (*p* < 0.001) against *B. cepacia* Bc23 causing at 4×MIC a killing rate of 58.5 ± 2.8% only. The effect against preformed biofilm was generally dose-dependent, especially in the case of *S. aureus* Sa2 and *B. cepacia* Bc23.

### TEM Observation Reveals Relevant Morphological Changes in *P. aeruginosa* Biofilm

**FIGURE 5:**
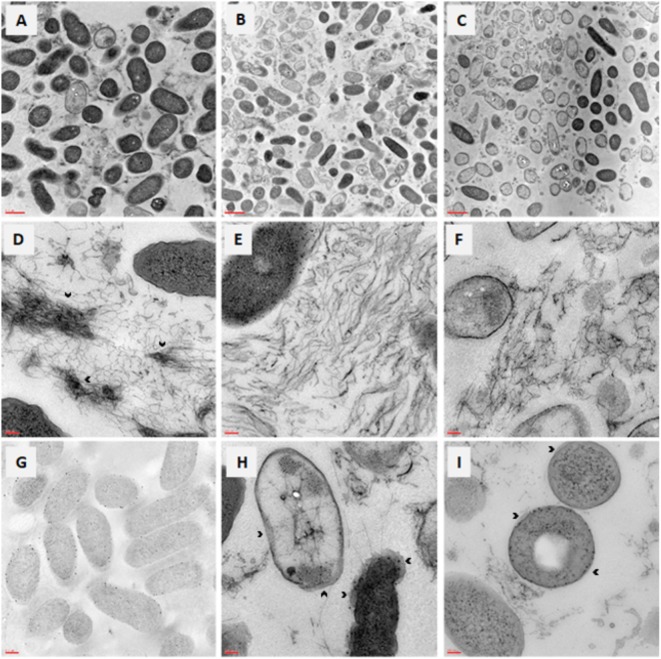
*In vitro* activity of AgNPs against preformed biofilm: TEM observation. Biofilm samples by *P. aeruginosa* DIN1 were allowed to form for 24 h in 96-well microtiter plate, and then exposed for further 24 h to **(A,D)** vehicle only (control), **(B,E,G–I)** AgNPs at 4×MIC, **(C,F)** Tobramycin at 4×MIC. Photograph **G** was taken without contrast to better highlight the periplasmic localization of AgNPs. The dense “tangle-like” organization of the extracellular polymeric substance biofilm matrix observed in control samples (as shown by the arrowheads in **D**), was significantly deconstructed following exposure to AgNPs **(E)** and, to a lesser extent, to Tobramycin **(F)**. Outer membrane separation is shown by the arrowheads in photographs **H,I**, whereas protein aggregation inside cells is evident in photograph **I**. Magnification: 7.000× **(A–C)**, 50.000× **(D–F,H,I)**, 20.000× **(G)**.

The effects caused on *P. aeruginosa* DIN1 mature biofilm by the exposure to AgNPs or Tobramycin at 4×MIC were evaluated by TEM observation (**Figure 5**). Most of the cells composing untreated control biofilm displayed an intact morphology - with regard to shape, wall, nucleoid, and inclusion body - and smooth surface (**Figure 5A**). Contrarily, biofilm cells treated for 24 h with AgNPs (**Figure 5B**) or Tobramycin (**Figure 5C**) revealed to be mostly killed, as indicated by relevant morphological changes in the cell wall and membrane corrugation damage. The EPS matrix, abundant and showing a complex “hank-like” organization in untreated biofilms (**Figure 5D**), resulted to be significantly deconstructed and quantitatively affected following exposure to AgNPs (**Figure 5E**) and, to a lesser extent, to Tobramycin (**Figure 5F**). Particularly, biofilm exposed to AgNPs presented evidence for lysis and emptying with low electron dense contrast inside, therefore suggesting a stress-response induced by exposure to AgNPs, probably consisting of changes in membrane polarization and/or permeability. Electron-dense AgNPs localized first in the periplasmic space, then moved to the cytoplasm in lysed cells (**Figures 5G–I**).

### AgNPs Are Not Toxic to *Galleria mellonella* Larvae

**FIGURE 6:**
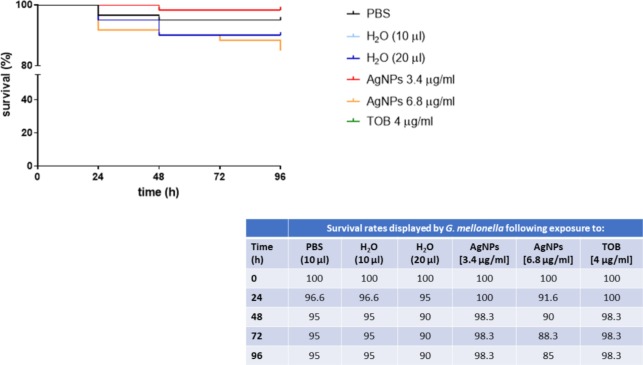
Survival curves for *Galleria mellonella* larvae against AgNPs and Tobramycin. Each data point represents the percentage survival (as detailed in the Table) of *G. mellonella* larvae (*n* = 20/group, repeated on three different occasions), following injection with 10 or 20 μl volumes of specific AgNPs (6.8 and 3.4 μg/ml), or Tobramycin (TOB) (4 μg/ml) concentrations, and incubation for 96 h at 37°C. Control larvae were injected with H_2_0 (vehicle), or PBS (to allow for the trauma associated with injection). Larvae were monitored daily for survival until 96 h.

AgNPs and Tobramycin were comparatively evaluated for toxicity in *G. mellonella*, and results are shown in **Figure 6**. Over the study period, the mortality rate ranged from 0 to 1.7% and from 8.3 to 11.7% following exposure to AgNPs, respectively, at 3.4 and 6.8 μg/ml, whereas no mortality was observed for Tobramycin. No significant differences were observed over time in mortality rate between control and treated larvae, regardless of AgNPs or Tobramycin concentrations tested, and found between AgNPs and Tobramycin.

## Discussion

Overall, the present study provides the experimental proof that AgNPs have the potential for an effective therapeutic agent for the treatment of lung infections caused by Gram-negative antibiotic-resistant pathogens in CF patients.

MIC and MBC values indicated good *in vitro* activity of AgNPs against all the strains studied, although with striking differences. In agreement with Zhang et al. (2015), we found that *P. aeruginosa* and *B. cepacia*, the most harmful pathogens in CF patients, were the most susceptible to AgNPs exposure, whereas a significantly lower activity was found against *S. aureus*. Time-kill studies showed that the effect against Gram-negative pathogens is bactericidal, dose-and time-dependent and quickly achieved, especially in the case of *S. maltophilia* whose complete killing was observed within 2 h-exposure at 1×MIC. Contrarily, only a bacteriostatic effect was observed in *S. aureus*. It is worth noting that against *P. aeruginosa* AgNPs exhibited a comparable, or even higher, antibacterial activity compared to Tobramycin, a first-line antibiotic for the therapy of CF *P. aeruginosa* infections. The difference in action of AgNPs against Gram-positive and Gram-negative bacteria might be attributed to the difference in their cell wall composition – particularly in peptidoglycan structure and membrane lipids contents – which acts as a barrier against penetration of NPs (Hajipour et al., 2012).

In chronically infected CF patients, pathogens adapt to the highly stressed CF lung environment by forming biofilm, a sessile mono- or poly-microbial community embedded in an EPS matrix and inherently resistant both to antibiotic therapy and host immune response. In particular, biofilm-mediated chronic *P. aeruginosa* infections represent the major factor leading to the increased morbidity rates and premature death seen in patients with CF (Høiby et al., 2010). We, therefore, sought to assess the activity of AgNPs at supra-inhibitory concentrations against already established biofilms. Our results indicated that AgNPs are able to reduce significantly the viability of mature biofilm formed by all species tested, although to different extents. The anti-biofilm activity was particularly relevant against biofilms preformed by *P. aeruginosa* and *S. aureus*, whose viability was reduced of at least 98% already at concentrations corresponding to 2×MIC (8.5 μg/ml). Interestingly, in the case of *P. aeruginosa*, the activity exhibited by AgNPs was comparable to that of Tobramycin. Loo et al. (2014), using AgNPs with an average diameter of 8–10 nm, yielded a reduction of *P. aeruginosa* biofilms of at least 90% only after exposure to the relevant concentration of 600 μg/ml, thus indicating the high effectiveness of AgNPs preparation we used in the present work. This is probable due to the greater purity of our AgNPs suspension that was synthesized in the absence of contaminants, such as inorganic or organic agents, commonly used as stabilizers in other preparations (Kittler et al., 2010). Our findings also showed that *S. maltophilia* and *B. cepacia* mature biofilms were less susceptible to AgNPs exposure, therefore warranting further studies to clarify the reasons underlying this specific activity against preformed biofilm.

Multiple mechanisms have been proposed to elucidate the killing of planktonic bacterial cells by AgNPs: disruption of cellular morphology, enzyme inactivation, inhibition of DNA replication, formation of reactive oxygen species, and generation of oxidative stress (Yamanaka et al., 2005; Li et al., 2011; Morones-Ramirez et al., 2013; Dizaj et al., 2014). Contrarily, the research focus on NPs-biofilm interactions is still in its early stages. A mini-review highlighting key physicochemical and biological processes that affect interactions and accumulation of NPs by bacterial biofilms has been recently published by Ikuma et al. (2015). To gain insights into the mechanisms underlying the relevant AgNPs activity we observed against preformed *P. aeruginosa* biofilm, we used TEM to evaluate the morphological effects occurring to the biofilm’s ultrastructure following exposure to AgNPs at lethal concentrations. Confirming the results obtained from viable cell count assays, most of the biofilm cells were dead as suggested by the relevant damage to the cell wall and membrane. The high efficiency in killing activity is probably due to the accumulation of NPs embedded in the biofilm (Ikuma et al., 2015), and to their inability to agglomerate thus make them more efficient in penetrating into the different extent of biofilm. The size cut-off for optimal penetration into *P. aeruginosa* biofilm clusters was previously located around 100–130 nm (Forier et al., 2014), and this is consistent with our results since the mean diameter of AgNPs we used was about 44 nm. We also observed the presence of high-density intracellular aggregates, suggestive of protein aggregation and probably due to protein misfolding secondary to the impairment of disulfide bond formation caused by silver ions (Morones-Ramirez et al., 2013). Furthermore, AgNPs exposure caused a drastic morphological change in the cell envelope, as shown by the outer membrane separation. These physical alterations in cell morphology might be suggestive for an increased outer membrane permeability, as also described for *Escherichia coli* (Morones-Ramirez et al., 2013). The EPS matrix is the primary emergent- and adaptive- property of the microbial cells forming a biofilm (Flemming and Wingender, 2010) and, therefore, it may potentially change in response to the presence of NPs. Confirming this, we observed that AgNPs exposure causes a significant reduction in the amount of EPS and its de-structuration, that is the lack of the “hank-like” organization observed in untreated *P. aeruginosa* biofilm. A similar effect, although to a lesser extent, was observed after exposure to Tobramycin. These findings are worth nothing since that EPS matrix – due to its high content in proteins, polysaccharides and nucleic acids – acts as a physicochemical barrier counteracting the antibiotic penetration through biofilm, with consequent loss in effectiveness of the antibiotic therapy and failure in the ultimate eradication of the infection. Most of the studies demonstrated the synergistic effect of AgNPs with antibiotics were focused against free-floating, planktonic, cells of Gram-positive and Gram-negative pathogens (Shahverdi et al., 2007; Singh et al., 2013; Panáček et al., 2016). As a whole, the morphological changes we observed at microscopic observation support the potential use of AgNPs as adjuvant also in the antibiotic therapy of biofilm-related infections, since NPs might: (i) increase the antibiotic susceptibility of drug-resistant cells by enhancing the membrane permeability to the antibiotic, and (ii) favor the antibiotic penetration through biofilm without being sequestered by EPS.

Although the antimicrobial activity of AgNPs has resulted in their use in various consumer products and medical applications, there is lack of information on their potential to induce adverse health effects. Although mammalian models are generally used to evaluate the toxicity of a compound, cheaper, time-saving, and ethically more acceptable invertebrate models of infection have been introduced (López Hernández et al., 2015). The innate immune system of these invertebrates is in fact functionally similar to that of mammals; furthermore, the virulence of many human pathogens is comparable in wax moth larvae and mammals (Tsai et al., 2016; Wojda, 2017). In the current study, cytotoxicity of AgNPs was evaluated in the larvae of the greater wax moth *Galleria mellonella*. Survival curves obtained following a single exposure of several doses indicated that, similarly to Tobramycin AgNPs remained non-toxic toward larvae up to the maximum concentration tested of 6.8 μg/ml, effective against both *B. cepacia* planktonic and biofilm cells, but against planktonic cells only in the case of *P. aeruginosa* and *S. maltophilia*. Due to technical reasons, it has not been possible to test cytotoxicity of AgNPs at higher concentrations. This could not be a limitation since previous *in vitro* and *in vivo* studies reported no toxic effect against lung epithelial cells up to 100 μg/ml (Miyayama and Matsuoka, 2016; Wiemann et al., 2017). However, Jeannet et al. (2016) have recently observed that exposure of well-differentiated CF human bronchial epithelium to aerosolized silver nanoaerosols (20 nm diameter) resulted in significantly higher necrosis and IL-8 secretion than normal HBE cells, although no functional and structural alterations of the epithelia were observed. Further, most of pharmacokinetic studies suggested that tissue distribution of AgNPs is size-dependent, regardless of the exposure route: smaller AgNPs (≤60 nm diameter) are mainly distributed to the liver, whereas larger ones (≥80 nm diameter) to the spleen (Lin et al., 2015). Particularly, the particle size and the tendency of particles to form agglomerates affect the distribution pathway in the lungs of rats (Takenaka et al., 2001). Future works aimed at evaluating the potential size-dependent toxicity of AgNPs should be carried out using both models and exposure modalities relevant to CF.

Using NPs as an antibacterial agent is a new and talented approach that has many advantages in comparison to conventional antimicrobial agents, such as low cost and simple NPs preparation, easy penetration into the bacterial cell or through matrix of biofilm communities due to NPs small dimensions, less time to kill bacteria, and lower probability to develop resistance due to the multiple and simultaneous mechanisms of NPs action (Rai et al., 2012; Iravani et al., 2014; Franci et al., 2015). Beside fighting bacterial resistance NPs can also act as a “medium and carrier” of antibiotics. The use of AgNPs combined with antimicrobial agents might help reduce the NP toxic potential, to avoid the potential for development of resistance and, above all, strongly enhance the microbicidal effect of antibiotics against both planktonic and biofilm cells (Rai et al., 2012; Habash et al., 2014; Iravani et al., 2014).

The *in vitro* antibacterial and anti-biofilm activity toward well-known CF pathogens, as well as low *in vivo* toxicity showed by AgNPs in the present study support the potential use of nanosized delivery systems for silver-based therapeutics aimed at reducing the antibiotic burden of patients treated for chronic infections. The therapeutic potential of AgNPs in CF patients will have to be confirmed by more comprehensive *in vitro* and *in vivo* studies performed under conditions relevant to CF lung – i.e., in the presence of CF sputum and under reduced oxygen tension - and aimed at: (i) confirming their activity against a higher number of strains; (ii) improving their antibacterial potential by acting on physicochemical properties (i.e., size, surface charge, and morphology); (iii) evaluating the potential toxicity associated with therapeutically effective concentrations and, finally (iv) assessing the rate of development of resistance. AgNPs might be tested as alone and combined with an antibiotic, for example preparing silica coated NPs embedded into a matrix of chitosan drugged with an antibiotic: silica may improve antibiotic dissolution, whereas chitosan could allow its entrapment for effective delivery and maintenance at the site of infection.

## Author Contributions

AP, TB, LS, and GDB designed the study. AP, CG, and DB performed the experiments and interpreted the data. AP and GDB wrote the manuscript. TB and LS contributed to the formulation of NPs. RR and AA provided editorial assistance.

## Conflict of Interest Statement

The authors declare that the research was conducted in the absence of any commercial or financial relationships that could be construed as a potential conflict of interest. The reviewers CC and LDM declared a shared affiliation, with no collaboration, with the authors to the handling Editor.

## Footnotes

1 https://clinicaltrials.gov/ct2/home

## Abbreviations

AgNPs: silver nanoparticles
CAMHB: cation-adjusted Muller-Hinton broth
CF: cystic fibrosis
EPS: extracellular polymeric substance
FE-SEM: field emission scanning electron microscopy
MHA: Muller-Hinton agar
OD: optical density
PBS: phosphate buffered saline
TSA: tryptone soya agar
TSB: tryptone soya broth

## References

[B1] Berdah L. Taytard J. Leyronnas S. Clement A. Boelle P. Y. Corvol H. (2018). *Stenotrophomonas maltophilia*: a marker of lung disease severity. *Pediatr. Pulmonol.* 53 426–430. 10.1002/ppul.23943 29314745 PMC5900908

[B2] Blaskovich M. A. Butler M. S. Cooper M. A. (2017). Polishing the tarnished silver bullet: the quest for new antibiotics. *Essays Biochem.* 61 103–114. 10.1042/EBC20160077 28258234 PMC5869247

[B3] Ciofu O. Hansen C. R. Høiby N. (2013). Respiratory bacterial infections in cystic fibrosis. *Curr. Opin. Pulm. Med.* 19 251–258. 10.1097/MCP.0b013e32835f1afc 23449384

[B4] Clinical Laboratory Standards Institute [CLSI] (2016). *Performance Standards for Antimicrobial Susceptibility Testing, M100S*, 26th Edn. Wayne, PA: CLSI.

[B5] Cox M. J. Allgaier M. Taylor B. Baek M. S. Huang Y. J. Daly R. A. (2010). Airway microbiota and pathogen abundance in age-stratified cystic fibrosis patients. *PLoS One* 5:e11044. 10.1371/journal.pone.0011044 20585638 PMC2890402

[B6] Cystic Fibrosis Foundation (2016). *Patient Registry Annual Data Report 2015.* Bethesda, MD: Cystic Fibrosis Foundation.

[B7] de la Fuente-Núñez C. Reffuveille F. Fernández L. Hancock R. E. (2013). Bacterial biofilm development as a multicellular adaptation: antibiotic resistance and new therapeutic strategies. *Curr. Opin. Microbiol.* 16 580–589. 10.1016/j.mib.2013.06.013 23880136

[B8] Desbois A. P. Coote P. J. (2012). Utility of greater wax moth larva (*Galleria mellonella*) for evaluating the toxicity and efficacy of new antimicrobial agents. *Adv. Appl. Microbiol.* 78 25–53. 10.1016/B978-0-12-394805-2.00002-6 22305092

[B9] Dizaj S. M. Lotfipour F. Barzegar-Jalali M. Zarrintan M. H. Adibkia K. (2014). Antimicrobial activity of the metals and metal oxide nanoparticles. *Mat. Sci. Eng. C Mater. Biol. Appl.* 44 278–284. 10.1016/j.msec.2014.08.031 25280707

[B10] Flemming H. C. Wingender J. (2010). The biofilm matrix. *Nat. Rev. Microbiol.* 8 623–633. 10.1038/nrmicro2415 20676145

[B11] Forier K. Messiaen A. S. Raemdonck K. Nelis H. De Smedt S. Demeester J. (2014). Probing the size limit for nanomedicine penetration into *Burkholderia multivorans* and *Pseudomonas aeruginosa* biofilms. *J. Control Release* 195 21–28. 10.1016/j.jconrel.2014.07.061 25125326

[B12] Franci G. Falanga A. Galdiero S. Palomba L. Rai M. Morelli G. (2015). Silver nanoparticles as potential antibacterial agents. *Molecules* 20 8856–8874. 10.3390/molecules20058856 25993417 PMC6272636

[B13] Habash M. B. Park A. J. Vis E. C. Harris R. J. Khursigara C. M. (2014). Synergy of silver nanoparticles and aztreonam against *Pseudomonas aeruginosa* PAO1 biofilms. *Antimicrob. Agents Chemother.* 58 5818–5830. 10.1128/AAC.03170-14 25049240 PMC4187931

[B14] Hajipour M. J. Fromm K. M. Ashkarran A. A. Jimenez de Aberasturi D. de Larramendi I. R. Rojo T. (2012). Antibacterial properties of nanoparticles. *Trends Biotechnol.* 30 499–511. 10.1016/j.tibtech.2012.06.004 22884769

[B15] Høiby N. Bjarnsholt T. Givskov M. Molin S. Ciofu O. (2010). Antibiotic resistance of bacterial biofilms. *Int. J. Antimicrob. Agents* 35 322–332. 10.1016/j.ijantimicag.2009.12.011 20149602

[B16] Hunt B. E. Weber A. Berger A. Ramsey B. Smith A. L. (1995). Macromolecular mechanisms of sputum inhibition of tobramycin activity. *Antimicrob. Agents Chemother.* 39 34–39. 10.1128/AAC.39.1.34 7535039 PMC162480

[B17] Ikuma K. Decho A. W. Lau B. L. (2015). When nanoparticles meet biofilms-interactions guiding the environmental fate and accumulation of nanoparticles. *Front. Microbiol.* 6:591. 10.3389/fmicb.2015.00591 26136732 PMC4468922

[B18] Iravani S. Korbekandi H. Mirmohammadi S. V. Zolfaghari B. (2014). Synthesis of silver nanoparticles: chemical, physical and biological methods. *Res. Pharm. Sci.* 9 385–406. 26339255 PMC4326978

[B19] Jain J. Arora S. Rajwade J. M. Omray P. Khandelwal S. Paknikar K. M. (2009). Silver nanoparticles in therapeutics: development of an antimicrobial gel formulation for topical use. *Mol. Pharm.* 6 1388–1401. 10.1021/mp900056g 19473014

[B20] Jeannet N. Fierz M. Schneider S. Künzi L. Baumlin N. Salathe M. (2016). Acute toxicity of silver and carbon nanoaerosols to normal and cystic fibrosis human bronchial epithelial cells. *Nanotoxicology* 10 279–291. 10.3109/17435390.2015.1049233 26011645

[B21] Johnson N. A. Southerland M. R. Youngs W. J. (2017). Recent developments in the medicinal applications of silver-NHC Complexes and imidazolium salts. *Molecules* 22:E1263. 10.3390/molecules22081263 28749425 PMC6152056

[B22] Kerem E. (2017). Cystic fibrosis: priorities and progress for future therapies. *Paediatr. Respir. Rev.* 24 14–16. 10.1016/j.prrv.2017.06.004 28697970

[B23] Kittler S. Greulich C. Diendorf J. Köller M. Epple M. (2010). Toxicity of silver nanoparticles increases during storage because of slow dissolution under release of silver ions. *Chem. Mater.* 22 4548–4554. 10.1021/cm100023p

[B24] Li W. R. Xie X. B. Shi Q. S. Duan S. S. Ouyang Y. S. Chen Y. B. (2011). Antibacterial effect of silver nanoparticles on *Staphylococcus aureus*. *Biometals* 24 135–141. 10.1007/s10534-010-9381-6 20938718

[B25] Lin Z. Monteiro-Riviere N. A. Riviere J. E. (2015). Pharmacokinetics of metallic nanoparticles. *WIREs Nanomed. Nanobiotechnol.* 7 189–217. 10.1002/wnan.1304 25316649

[B26] Loo C. Y. Young P. M. Cavaliere R. Whitchurch C. B. Lee W. H. Rohanizadeh R. (2014). Silver nanoparticles enhance *Pseudomonas aeruginosa* PAO1 biofilm detachment. *Drug Dev. Ind. Pharm.* 40 719–729. 10.3109/03639045.2013.780182 23594297

[B27] López Hernández Y. Yero D. Pinos-Rodríguez J. M. Gibert I. (2015). Animals devoid of pulmonary system as infection models in the study of lung bacterial pathogens. *Front. Microbiol.* 6:38. 10.3389/fmicb.2015.00038 25699030 PMC4316775

[B28] Magiorakos A. P. Srinivasan A. Carey R. B. Carmeli Y. Falagas M. E. Giske C. G. (2012). Multidrug-resistant, extensively drug-resistant and pandrug-resistant bacteria: an international expert proposal for interim standard definitions for acquired resistance. *Clin. Microbiol. Infect.* 18 268–281. 10.1111/j.1469-0691.2011.03570.x 21793988

[B29] Miyayama T. Matsuoka M. (2016). Involvement of lysosomal dysfunction in silver nanoparticle-induced cellular damage in A549 human lung alveolar epithelial cells. *J. Occup. Med. Toxicol.* 11:1. 10.1186/s12995-016-0090-0 26759602 PMC4709927

[B30] Morones-Ramirez J. R. Winkler J. A. Spina C. S. Collins J. J. (2013). Silver enhances antibiotic activity against gram-negative bacteria. *Sci. Transl. Med.* 5:190ra81. 10.1126/scitranslmed.3006276 23785037 PMC3771099

[B31] Olsen I. (2015). Biofilm-specific antibiotic tolerance and resistance. *Eur. J. Clin. Microbiol. Infect. Dis.* 34 877–886. 10.1007/s10096-015-2323-z 25630538

[B32] Panáček A. Smékalová M. Večeřová R. Bogdanová K. Röderová M. Kolář M. (2016). Silver nanoparticles strongly enhance and restore bactericidal activity of inactive antibiotics against multiresistant *Enterobacteriaceae*. *Colloids Surf. B. Biointerfaces* 142 392–399. 10.1016/j.colsurfb.2016.03.007 26970828

[B33] Pompilio A. Crocetta V. Pomponio S. Fiscarelli E. Di Bonaventura G. (2015). In vitro activity of colistin against biofilm by *Pseudomonas aeruginosa* is significantly improved under "cystic fibrosis-like" physicochemical conditions. *Diagn. Microbiol. Infect. Dis.* 82 318–325. 10.1016/j.diagmicrobio.2015.01.006 26004353

[B34] Rai M. K. Deshmukh S. D. Ingle A. P. Gade A. K. (2012). Silver nanoparticles: the powerful nanoweapon against multidrug-resistant bacteria. *J. Appl. Microbiol.* 112 841–852. 10.1111/j.1365-2672.2012.05253.x 22324439

[B35] Scotti L. Angelini A. Gasbarri C. Bucciarelli T. (2017). Uncoated negatively charged silver nanoparticles: speeding up the electrochemical synthesis. *Mater. Res. Express* 4:105001 10.1088/2053-1591/aa8c39

[B36] Shahverdi A. R. Fakhimi A. Shahverdi H. R. Minaian S. (2007). Synthesis and effect of silver nanoparticles on the antibacterial activity of different antibiotics against *Staphylococcus aureus* and *Escherichia coli*. *Nanomedicine* 3 168–171. 10.1016/j.nano.2007.02.001 17468052

[B37] Singh R. Wagh P. Wadhwani S. Gaidhani S. Kumbhar A. Bellare J. (2013). Synthesis, optimization, and characterization of silver nanoparticles from *Acinetobacter calcoaceticus* and their enhanced antibacterial activity when combined with antibiotics. *Int. J. Nanomedicine* 8 4277–4290. 10.2147/IJN.S48913 24235826 PMC3826770

[B38] Smith W. D. Bardin E. Cameron L. Edmondson C. L. Farrant K. V. Martin I. (2017). Current and future therapies for *Pseudomonas aeruginosa* infection in patients with cystic fibrosis. *FEMS Microbiol. Lett.* 364:fnx121. 10.1093/femsle/fnx121 28854668

[B39] Sondi I. Salopek-Sondi B. (2004). Silver nanoparticles as antimicrobial agent: a case study on *Escherichia coli* as a model for Gram-negative bacteria. *J. Colloid Interface Sci.* 275 177–182. 10.1016/j.jcis.2004.02.012 15158396

[B40] Speert D. P. Henry D. Vandamme P. Corey M. Mahenthiralingam E. (2002). Epidemiology of Burkholderia cepacia complex in patients with cystic fibrosis, Canada. *Emerg. Infect. Dis.* 8 181–187. 10.3201/eid0802.010163 11897071 PMC3369581

[B41] Stepanović S. Vuković D. Hola V. Di Bonaventura G. Djukić S. Cirković I. (2007). Quantification of biofilm in microtiter plates: overview of testing conditions and practical recommendations for assessment of biofilm production by staphylococci. *APMIS* 115 891–899. 10.1111/j.1600-0463.2007.apm_630.x 17696944

[B42] Takenaka S. Karg E. Roth C. Schulz H. Ziesenis A. Heinzmann U. (2001). Pulmonary and systemic distribution of inhaled ultrafine silver particles in rats. *Environ. Health Perspect.* 109 (Suppl. 4), 547–551. 10.1289/ehp.01109s4547 11544161 PMC1240579

[B43] Tsai C. J. Loh J. M. Proft T. (2016). *Galleria mellonella* infection models for the study of bacterial diseases and for antimicrobial drug testing. *Virulence* 7 214–229. 10.1080/21505594.2015.1135289 26730990 PMC4871635

[B44] Vimbela G. V. Ngo S. M. Fraze C. Yang L. Stout D. A. (2017). Antibacterial properties and toxicity from metallic nanomaterials. *Int. J. Nanomedicine* 12 3941–3965. 10.2147/IJN.S134526 28579779 PMC5449158

[B45] Wiemann M. Vennemann A. Blaske F. Sperling M. Karst U. (2017). Silver nanoparticles in the lung: toxic effects and focal accumulation of silver in remote organs. *Nanomaterials* 7:441. 10.3390/nano7120441 29231883 PMC5746931

[B46] Wojda I. (2017). Immunity of the greater wax moth *Galleria mellonella*. *Insect Sci.* 24 342–357. 10.1111/1744-7917.12325 26847724

[B47] Yamanaka M. Hara K. Kudo J. (2005). Bactericidal actions of a silver ion solution on *Escherichia coli* studied by energy filtering transmission electron microscopy and proteomic analysis. *Appl. Environ. Microbiol.* 71 7589–7593. 10.1128/AEM.71.11.7589-7593.2005 16269810 PMC1287701

[B48] Zhang F. Smolen J. A. Zhang S. Li R. Shah P. N. Cho S. (2015). Degradable polyphosphoester-based silver-loaded nanoparticles as therapeutics for bacterial lung infections. *Nanoscale* 7 2265–2270. 10.1039/c4nr07103d 25573163

