# Supplementary material for: Electrochemically Synthesized Silver Nanoparticles Are Active Against Planktonic and Biofilm Cells of Pseudomonas aeruginosa and Other Cystic Fibrosis-Associated Bacterial Pathogens
Source: Front Microbiol. 2018 Jul 5;9:1349. doi: 10.3389/fmicb.2018.01349 (PMC6041389; doi:10.3389/fmicb.2018.01349)
Supplement: Supplementary file 2 [file Data_Sheet_1.PDF]

Brookhaven Instruments Corp.
ZetaPals Particle Sizing Software Ver. 3.86

Date: Aug 4, 2017
Time: 12:16:56
Batch: 0

Sample ID **AgNPs-3W-cost-01-08-17-centr- (Combined)**
Operator ID **Luca**
Notes **04-08-2017**

Measurement Parameters:

Temperature	= 25.0 deg. C	Runs Completed	= 5
Liquid	= Water	Run Duration	= 00:01:00
Viscosity	= 0.890 cP	Total Elapsed Time	= 00:05:00
Ref.Index Fluid	= 1.330	Average Count Rate	= 274.3 kcps
Angle	= 90.00	Ref.Index Real	= 1.000
Wavelength	= 660.0 nm	Ref.Index Imag	= 0.000
Baseline	= Auto (Slope Analysis)	Dust Filter	= Off

AgNPs-3W-cost-01-08-17-centr- (Combined)

Effective Diameter: 30.1 nm
Polydispersity: 0.139
Baseline Index: 7.9
Elapsed Time: 00:05:00

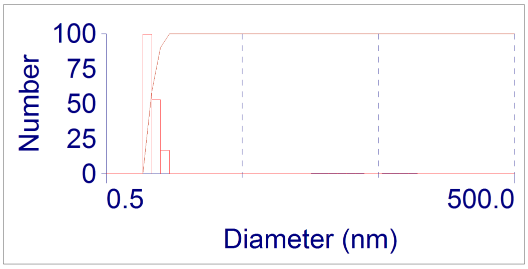

Multimodal Size Distribution

Run	Eff. Diam. (nm)	Half Width (nm)	Polydispersity	Baseline Index
1	29.1	11.1	0.145	7.5
2	30.0	9.7	0.105	7.1
3	29.9	12.3	0.168	9.0
4	30.6	11.5	0.140	7.8
5	30.9	12.0	0.150	8.3
Mean	30.1	11.3	0.142	7.9
Std. Error	0.3	0.4	0.010	0.3
Combined	30.1	11.2	0.139	7.9

Brookhaven Instruments Corp.
ZetaPals Particle Sizing Software Ver. 3.86

Date: Aug 4, 2017
Time: 12:16:56
Batch: 0

Sample ID **AgNPs-3W-cost-01-08-17-centr- (Combined)**
Operator ID **Luca**
Notes **04-08-2017**

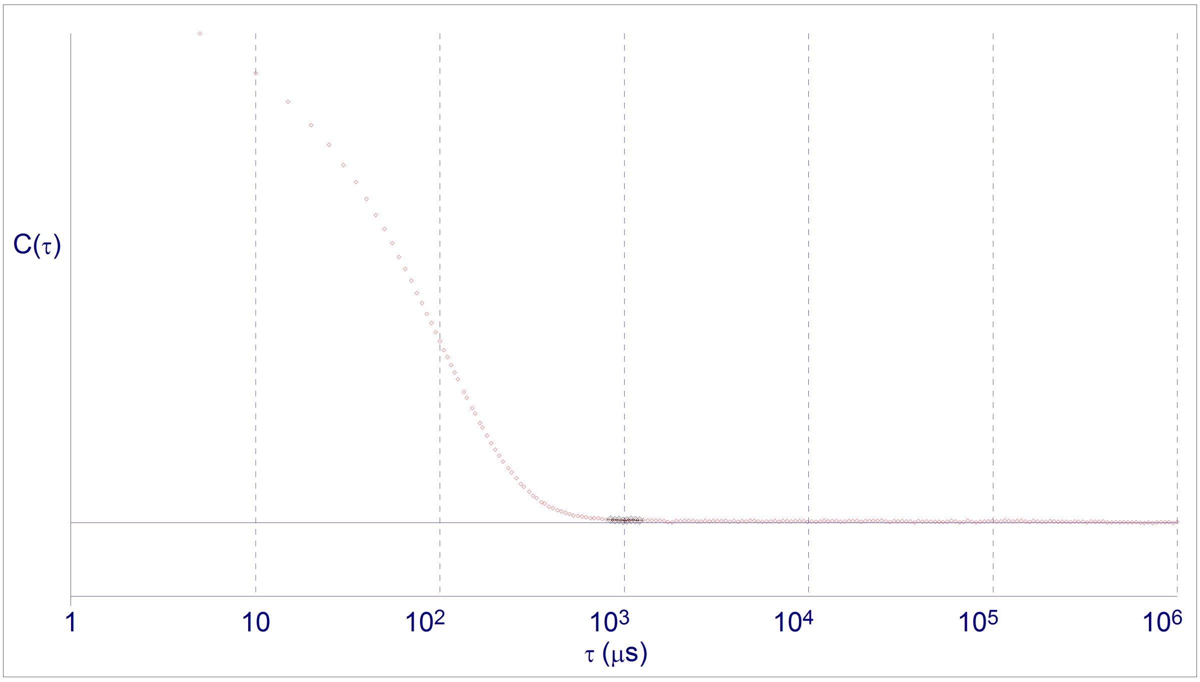

Correlation Function

Brookhaven Instruments Corp.
ZetaPals Particle Sizing Software Ver. 3.86

Date: Aug 4, 2017
Time: 12:16:56
Batch: 0

Sample ID **AgNPs-3W-cost-01-08-17-centr- (Combined)**
Operator ID **Luca**
Notes **04-08-2017**

Elapsed Time	00:05:00
Eff. Diam.	30.1 nm
Mean Diam.	32.1 nm
Polydispersity	0.139
GSD	1.435

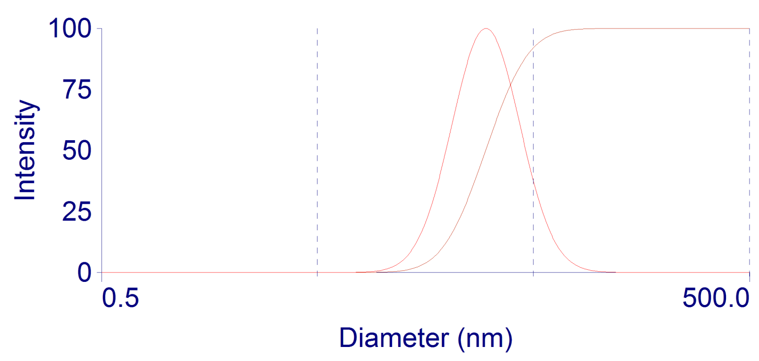

Lognormal Size Distribution

r(nm)	G(r)	C(r)	d(nm)	G(d)	C(d)	d(nm)	G(d)	C(d)
16.6	26	5	27.5	97	40	38.4	80	75
18.9	44	10	28.8	99	45	40.8	70	80
20.7	58	15	30.1	100	50	43.8	58	85
22.2	70	20	31.5	99	55	47.8	44	90
23.6	80	25	33.0	97	60	54.6	26	95
24.9	87	30	34.6	93	65			
26.2	93	35	36.4	87	70			

Brookhaven Instruments Corp.
ZetaPals Particle Sizing Software Ver. 3.86

Date: Aug 4, 2017
Time: 12:16:56
Batch: 0

Sample ID **AgNPs-3W-cost-01-08-17-centr- (Combined)**
Operator ID **Luca**
Notes **04-08-2017**

Elapsed Time	00:05:00
Mean Diam.	1.1 nm
Rel. Var.	0.011
Skew	2.134

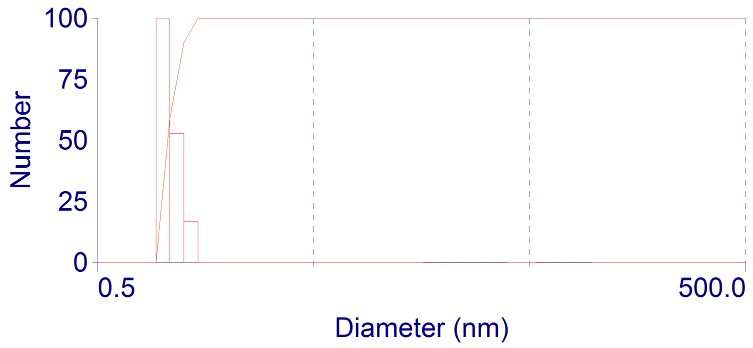

Multimodal Size Distribution

d(nm)	G(d)	C(d)	d(nm)	G(d)	C(d)	d(nm)	G(d)	C(d)
1.0	100	59	5.2	0	100	26.9	0	100
1.2	53	90	6.0	0	100	31.3	0	100
1.3	17	100	7.0	0	100	36.4	0	100
1.6	0	100	8.1	0	100	42.2	0	100
1.8	0	100	9.4	0	100	49.0	0	100
2.1	0	100	11.0	0	100	57.0	0	100
2.5	0	100	12.7	0	100	66.2	0	100
2.9	0	100	14.8	0	100	76.9	0	100
3.3	0	100	17.2	0	100	89.3	0	100
3.8	0	100	20.0	0	100	103.7	0	100
4.5	0	100	23.2	0	100	120.4	0	100

Brookhaven Instruments Corp.
ZetaPals Particle Sizing Software Ver. 3.86

Date: Aug 4, 2017
Time: 12:16:56
Batch: 0

Sample ID **AgNPs-3W-cost-01-08-17-centr- (Combined)**
Operator ID **Luca**
Notes **04-08-2017**

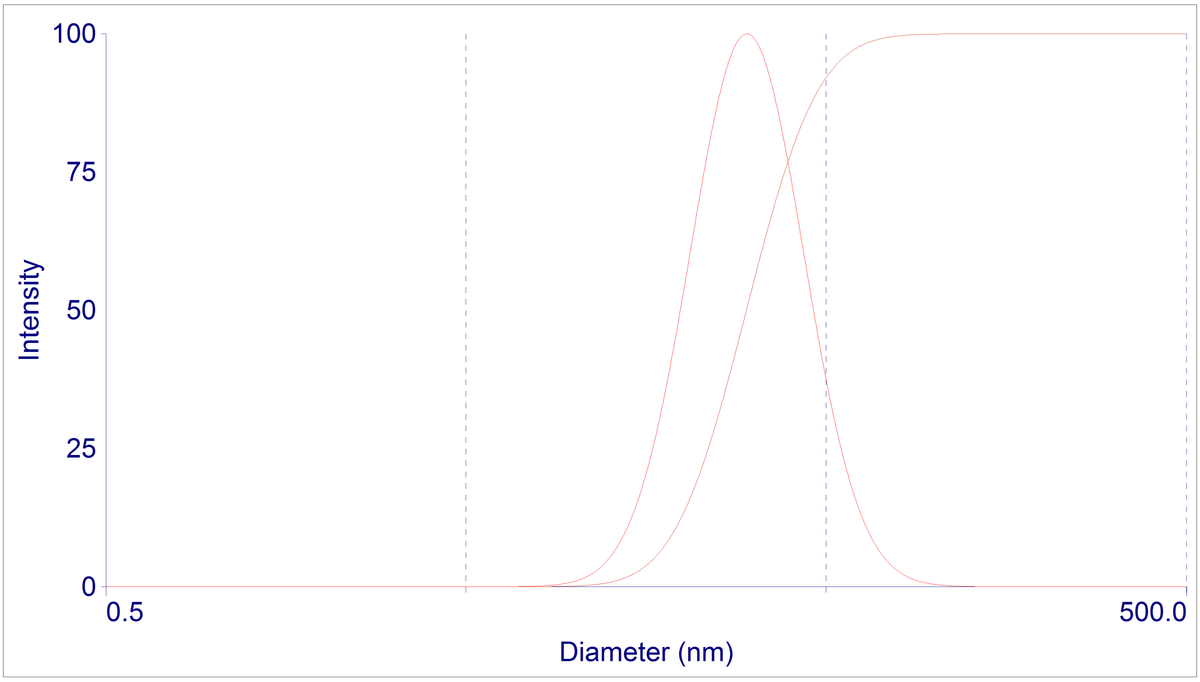

Lognormal Distribution

Brookhaven Instruments Corp.
ZetaPals Particle Sizing Software Ver. 3.86

Date: Aug 4, 2017
Time: 12:16:56
Batch: 0

Sample ID **AgNPs-3W-cost-01-08-17-centr- (Combined)**
Operator ID **Luca**
Notes **04-08-2017**

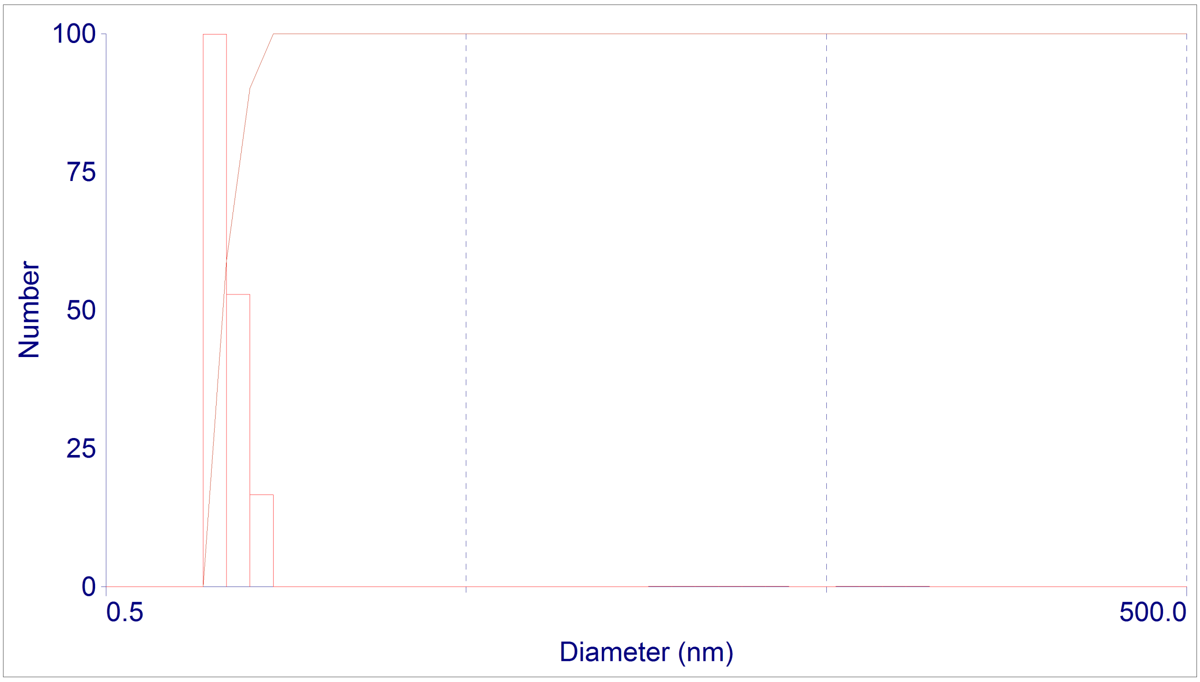

Multimodal Size Distribution
