# Supplementary material for: Electrochemically Synthesized Silver Nanoparticles Are Active Against Planktonic and Biofilm Cells of Pseudomonas aeruginosa and Other Cystic Fibrosis-Associated Bacterial Pathogens
Source: Front Microbiol. 2018 Jul 5;9:1349. doi: 10.3389/fmicb.2018.01349 (PMC6041389; doi:10.3389/fmicb.2018.01349)
Supplement: Supplementary file 3 [file Data_Sheet_2.PDF]

Brookhaven Instruments Corp.
ZetaPals Particle Sizing Software Ver. 3.86

Date: Feb 2, 2018
Time: 09:11:22
Batch: 0

Sample ID **AgNPs-Hellma-CUV-plastica (Combined)**
Operator ID **Luca**
Notes **2018-02-02**

Measurement Parameters:

Temperature	= 25.0 deg. C	Runs Completed	= 5
Liquid	= Water	Run Duration	= 00:01:00
Viscosity	= 0.890 cP	Total Elapsed Time	= 00:05:00
Ref.Index Fluid	= 1.330	Average Count Rate	= 315.4 kcps
Angle	= 90.00	Ref.Index Real	= 1.000
Wavelength	= 660.0 nm	Ref.Index Imag	= 0.000
Baseline	= Auto (Slope Analysis)	Dust Filter	= Off

AgNPs-Hellma-CUV-plastica (Combined)

Effective Diameter: 32.7 nm
Polydispersity: 0.219
Baseline Index: 9.2
Elapsed Time: 00:05:00

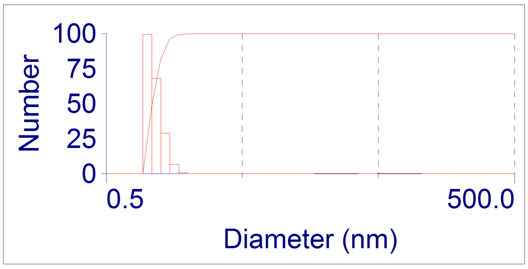

Multimodal Size Distribution

Run	Eff. Diam. (nm)	Half Width (nm)	Polydispersity	Baseline Index
1	32.9	15.6	0.224	8.8
2	33.2	15.6	0.220	8.7
3	33.2	15.8	0.227	9.4
4	32.5	15.1	0.215	9.8
5	31.7	14.4	0.208	9.5
Mean	32.7	15.3	0.219	9.2
Std. Error	0.3	0.2	0.003	0.2
Combined	32.7	15.3	0.219	9.2

Brookhaven Instruments Corp.
ZetaPals Particle Sizing Software Ver. 3.86

Date: Feb 2, 2018
Time: 09:11:22
Batch: 0

Sample ID **AgNPs-Hellma-CUV-plastica (Combined)**
Operator ID **Luca**
Notes **2018-02-02**

Elapsed Time	00:05:00
Eff. Diam.	32.7 nm
Mean Diam.	36.1 nm
Polydispersity	0.219
GSD	1.560

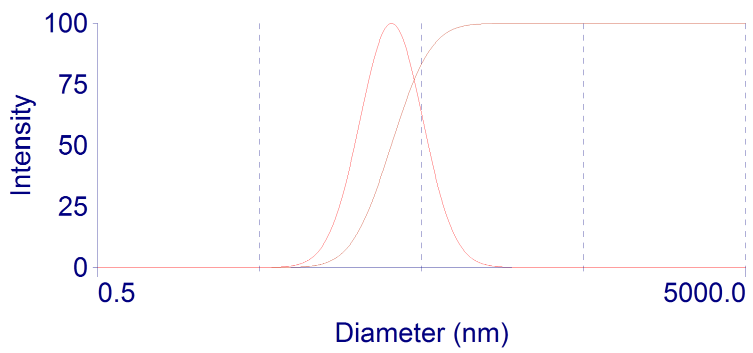

Lognormal Size Distribution

r(nm)	G(r)	C(r)	d(nm)	G(d)	C(d)	d(nm)	G(d)	C(d)
15.7	26	5	29.2	97	40	44.1	80	75
18.5	44	10	30.9	99	45	47.5	70	80
20.6	58	15	32.7	100	50	51.8	58	85
22.5	70	20	34.6	99	55	57.8	44	90
24.2	80	25	36.6	97	60	67.9	26	95
25.9	87	30	38.8	93	65			
27.5	93	35	41.3	87	70			

Brookhaven Instruments Corp.
ZetaPals Particle Sizing Software Ver. 3.86

Date: Feb 2, 2018
Time: 09:11:22
Batch: 0

Sample ID **AgNPs-Hellma-CUV-plastica (Combined)**
Operator ID **Luca**
Notes **2018-02-02**

Elapsed Time	00:05:00
Mean Diam.	1.1 nm
Rel. Var.	0.019
Skew	1.574

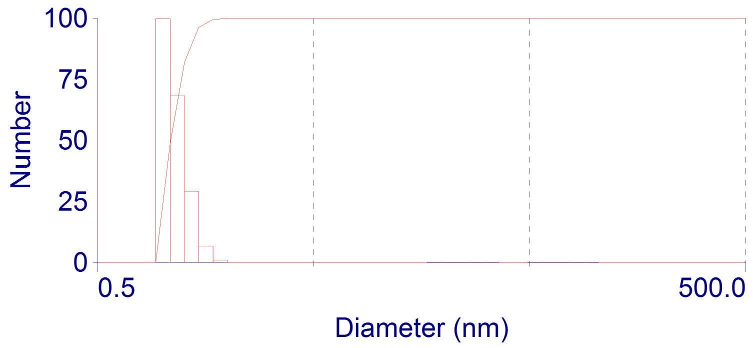

Multimodal Size Distribution

d(nm)	G(d)	C(d)	d(nm)	G(d)	C(d)	d(nm)	G(d)	C(d)
1.0	100	49	5.3	0	100	28.5	0	100
1.2	68	82	6.2	0	100	33.2	0	100
1.4	29	96	7.2	0	100	38.7	0	100
1.6	7	100	8.4	0	100	45.0	0	100
1.8	1	100	9.8	0	100	52.4	0	100
2.1	0	100	11.4	0	100	61.0	0	100
2.5	0	100	13.3	0	100	71.1	0	100
2.9	0	100	15.5	0	100	82.8	0	100
3.4	0	100	18.1	0	100	96.4	0	100
3.9	0	100	21.0	0	100	112.2	0	100
4.6	0	100	24.5	0	100	130.7	0	100

Brookhaven Instruments Corp.
ZetaPals Particle Sizing Software Ver. 3.86

Date: Feb 2, 2018
Time: 09:11:22
Batch: 0

Sample ID **AgNPs-Hellma-CUV-plastica (Combined)**
Operator ID **Luca**
Notes **2018-02-02**

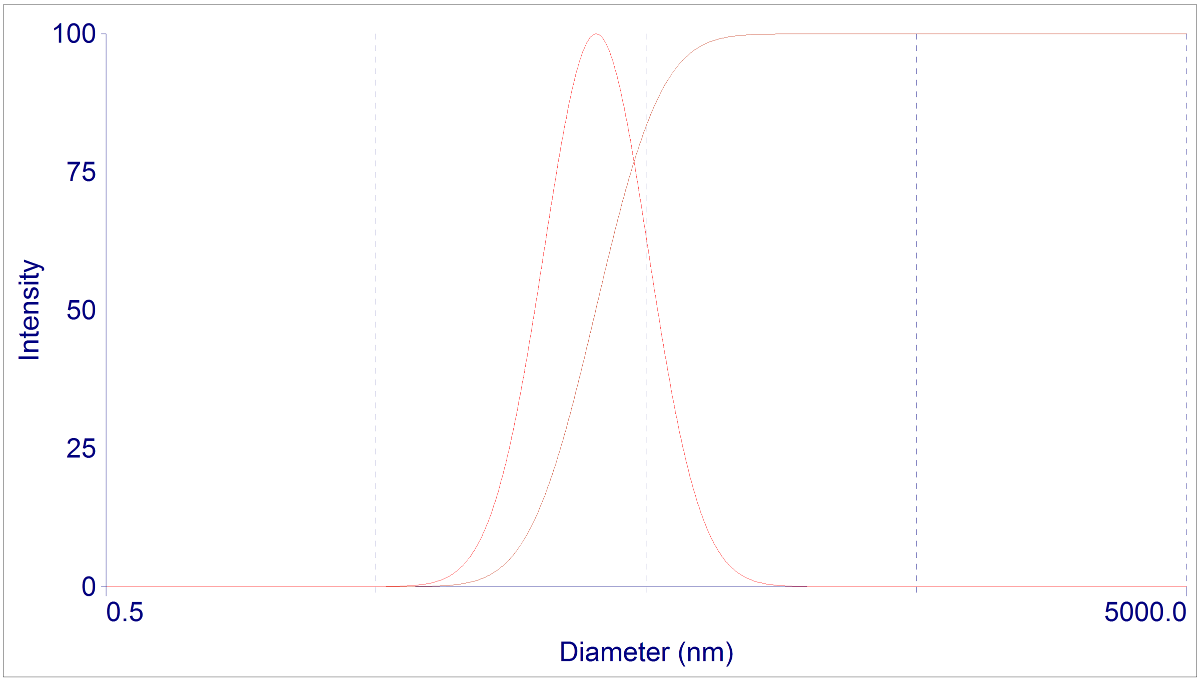

Lognormal Distribution

Brookhaven Instruments Corp.
ZetaPals Particle Sizing Software Ver. 3.86

Date: Feb 2, 2018
Time: 09:11:22
Batch: 0

Sample ID **AgNPs-Hellma-CUV-plastica (Combined)**
Operator ID **Luca**
Notes **2018-02-02**

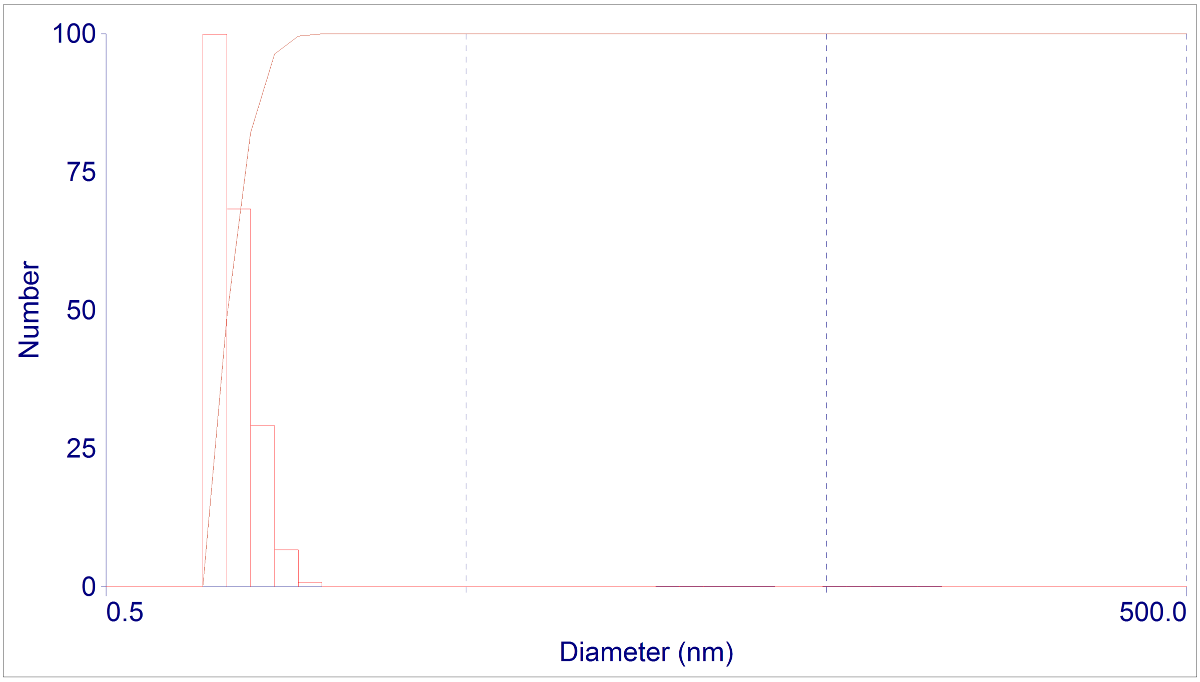

Multimodal Size Distribution

Brookhaven Instruments Corp.
ZetaPals Particle Sizing Software Ver. 3.86

Date: Feb 2, 2018
Time: 09:11:22
Batch: 0

Sample ID **AgNPs-Hellma-CUV-plastica (Combined)**
Operator ID **Luca**
Notes **2018-02-02**

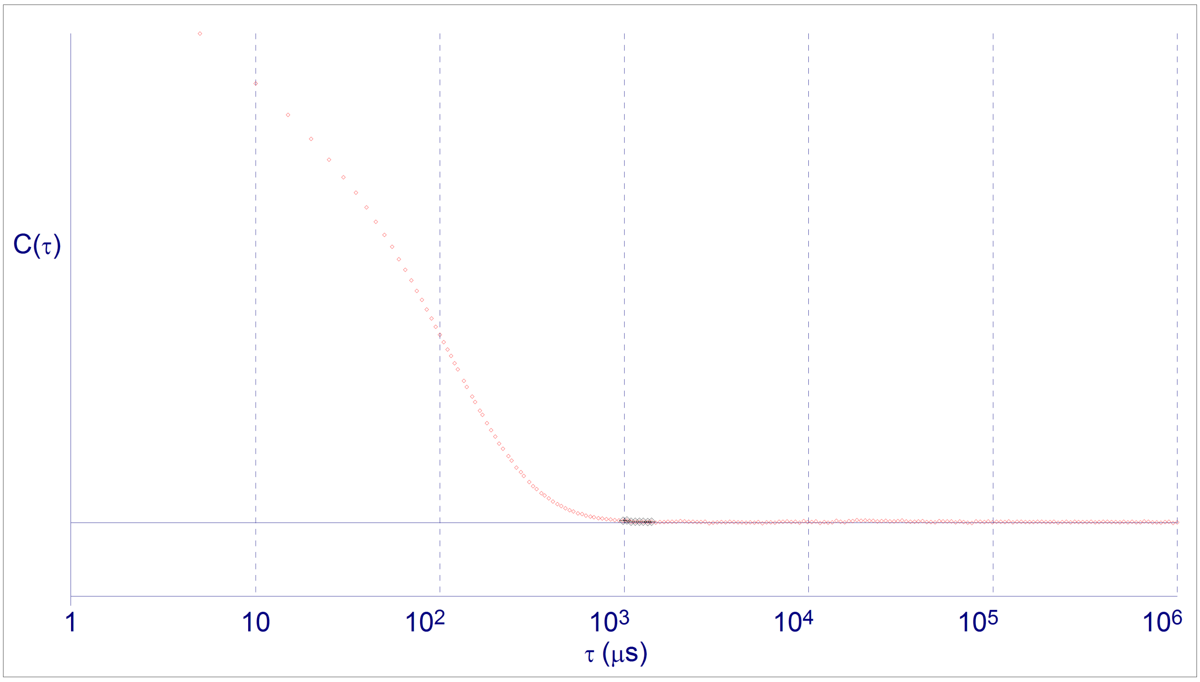

Correlation Function
