# Supplementary material for: Electrochemically Synthesized Silver Nanoparticles Are Active Against Planktonic and Biofilm Cells of Pseudomonas aeruginosa and Other Cystic Fibrosis-Associated Bacterial Pathogens
Source: Front Microbiol. 2018 Jul 5;9:1349. doi: 10.3389/fmicb.2018.01349 (PMC6041389; doi:10.3389/fmicb.2018.01349)
Supplement: Supplementary file 4 [file Data_Sheet_3.PDF]

Brookhaven Instruments Corp.
PALS Zeta Potential Analyzer Ver. 3.52

Date: Feb 2, 2018
Time: 09:33:24
Batch: 1

Sample ID **AgNPs-Hellma-CUV-plastica-H (Combined)**
Operator ID **Luca**
Notes **2018-02-02**

Measurement Parameters:

Mean Zeta Potential	= -37.83 mV	Liquid	= Water
Zeta Potential Model	= Hückel	Temperature	= 25.0 °C
Mean Mobility	= -1.97 (μ/s) / (V/cm)	Viscosity	= 0.890 cP
pH	= 7.40	Refractive Index	= 1.330
Conductance	= 71 μS	Dielectric Constant	= 78.54
Concentration	= 1.00 mg/mL	Particle Size	= 32.0 nm

Instrument Parameters:

Sample Count Rate	= 635 kcps	Voltage	= 4.00 volts
Ref. Count Rate	= 3122 kcps	Electric Field	= 10.81 V/cm
Wavelength	= 660.0 nm	User1	= 0.00
Field Frequency	= 2.00 Hz	User2	= 0.00
Cycles Per Run	= 10		

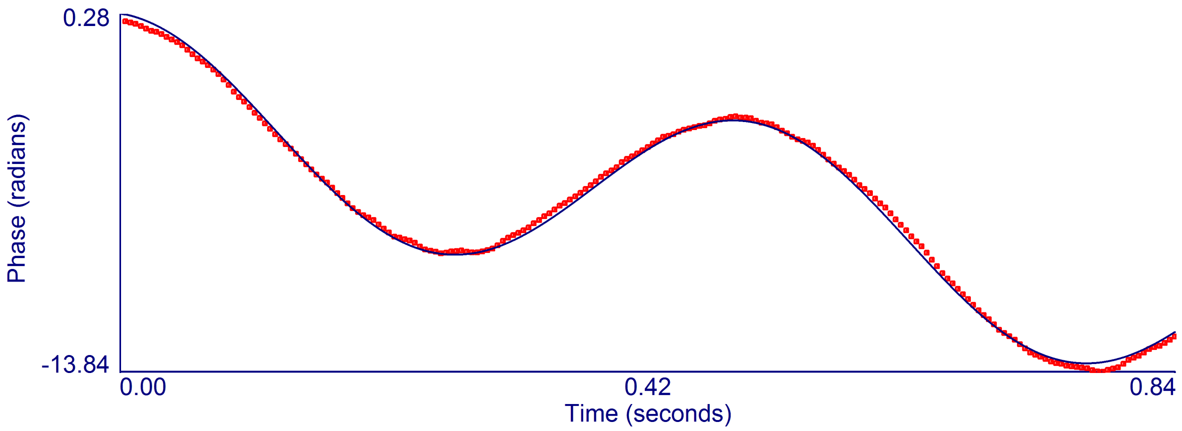

AgNPs-Hellma-CUV-plastica-H (Combined)

Run	Mobility	Zeta Potential (mV)	Rel. Residual
~~1~~	~~-1.76~~	~~-33.79~~	~~0.0155~~
2	-2.02	-38.79	0.0283
3	-1.83	-35.07	0.0255
4	-2.12	-40.76	0.0289
5	-2.06	-39.61	0.0164
~~6~~	~~-1.76~~	~~-33.87~~	~~0.0232~~
7	-1.85	-35.46	0.0353
8	-1.87	-35.81	0.0185
9	-2.10	-40.35	0.0242
10	-1.92	-36.84	0.0263
Mean	-1.97	-37.83	0.0254
Std. Error	0.04	0.82	0.0021
Combined	-1.96	-37.70	0.0121
