# Supplementary material for: Electrochemically Synthesized Silver Nanoparticles Are Active Against Planktonic and Biofilm Cells of Pseudomonas aeruginosa and Other Cystic Fibrosis-Associated Bacterial Pathogens
Source: Front Microbiol. 2018 Jul 5;9:1349. doi: 10.3389/fmicb.2018.01349 (PMC6041389; doi:10.3389/fmicb.2018.01349)
Supplement: Supplementary file 5 [file Data_Sheet_4.PDF]

Brookhaven Instruments Corp.
PALS Zeta Potential Analyzer Ver. 3.52

Date: Aug 4, 2017
Time: 12:55:36
Batch: 1

Sample ID **AgNPs-3W-cost-01-08-17-centr- (Combined)**
Operator ID **Luca**
Notes **04-08-2017**

Measurement Parameters:

Mean Zeta Potential	= -35.22 mV	Liquid	= Water
Zeta Potential Model	= Hückel	Temperature	= 25.0 °C
Mean Mobility	= -1.83 (μ/s) / (V/cm)	Viscosity	= 0.890 cP
pH	= 7.80	Refractive Index	= 1.330
Conductance	= 78 μS	Dielectric Constant	= 78.54
Concentration	= 1.00 mg/mL	Particle Size	= 111.0 nm

Instrument Parameters:

Sample Count Rate	= 146 kcps	Voltage	= 4.00 volts
Ref. Count Rate	= 2484 kcps	Electric Field	= 9.84 V/cm
Wavelength	= 660.0 nm	User1	= 0.00
Field Frequency	= 2.00 Hz	User2	= 0.00
Cycles Per Run	= 5		

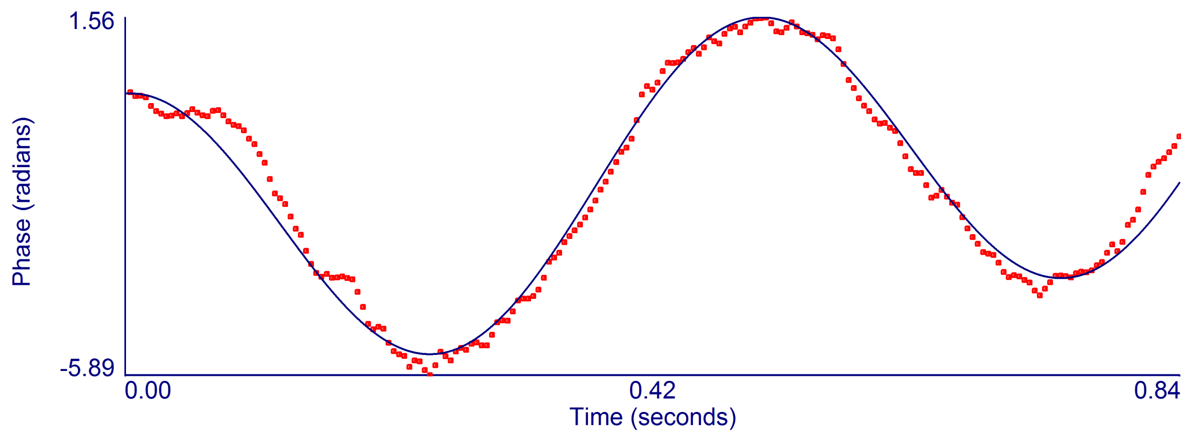

AgNPs-3W-cost-01-08-17-centr- (Combined)

Run	Mobility	Zeta Potential (mV)	Rel. Residual
1	-2.30	-44.23	0.0300
~~2~~	~~-0.90~~	~~-17.31~~	~~0.0565~~
3	-1.17	-22.40	0.0512
4	-2.06	-39.54	0.0549
5	-1.81	-34.69	0.0483
Mean	-1.83	-35.22	0.0461
Std. Error	0.24	4.69	0.0055
Combined	-1.83	-35.06	0.0254
